# SRC kinase-mediated signaling pathways and targeted therapies in breast cancer

**DOI:** 10.1186/s13058-022-01596-y

**Published:** 2022-12-29

**Authors:** Juan Luo, Hailin Zou, Yibo Guo, Tongyu Tong, Liping Ye, Chengming Zhu, Liang Deng, Bo Wang, Yihang Pan, Peng Li

**Affiliations:** 1 Scientific Research Center, The Seventh Affiliated Hospital of Sun Yat-Sen University, No. 628 Zhenyuan Road, Shenzhen, 518107 Guangdong People’s Republic of China; 2 Department of Urology, Pelvic Floor Disorders Center, The Seventh Affiliated Hospital of Sun Yat-Sen University, No. 628 Zhenyuan Road, Shenzhen, 518107 Guangdong People’s Republic of China; 3 Department of General Surgery, The Seventh Affiliated Hospital of Sun Yat-Sen University, No. 628 Zhenyuan Road, Shenzhen, 518107 Guangdong People’s Republic of China; 4 Department of Oncology, The Seventh Affiliated Hospital of Sun Yat-Sen University, No. 628 Zhenyuan Road, Shenzhen, 518107 Guangdong People’s Republic of China; 5 Guangdong Provincial Key Laboratory of Digestive Cancer Research, The Seventh Affiliated Hospital of Sun Yat-Sen University, No. 628 Zhenyuan Road, Shenzhen, 518107 Guangdong People’s Republic of China

**Keywords:** Breast cancer, SRC kinase, Signaling transduction, Tyrosine phosphorylation, Targeted therapy

## Abstract

Breast cancer (BC) has been ranked the most common malignant tumor throughout the world and is also a leading cause of cancer-related deaths among women. SRC family kinases (SFKs) belong to the non-receptor tyrosine kinase (nRTK) family, which has eleven members sharing similar structure and function. Among them, SRC is the first identified proto-oncogene in mammalian cells. Oncogenic overexpression or activation of SRC has been revealed to play essential roles in multiple events of BC progression, including tumor initiation, growth, metastasis, drug resistance and stemness regulations. In this review, we will first give an overview of SRC kinase and SRC-relevant functions in various subtypes of BC and then systematically summarize SRC-mediated signaling transductions, with particular emphasis on SRC-mediated substrate phosphorylation in BC. Furthermore, we will discuss the progress of SRC-based targeted therapies in BC and the potential future direction.

## Introduction

According to the global cancer statistics in 2020, female BC has surpassed lung cancer and been ranked as the most commonly diagnosed cancer in the world, with an estimated 2.3 million new cases and accounting for 11.7% of the total cases [1]. Early diagnosis and the continued improvement in treatment regiments, including surgery, radiotherapy, chemotherapy and biotherapy, have significantly improved the cure rates of patients with localized and some of the metastatic BCs [2]. However, BC is still the leading cause of cancer-related deaths among women worldwide, with 1/4 cancer cases and 1/6 cancer deaths [1]. Based on the expression of estrogen receptor (ER), progesterone receptor (PR) and human epidermal growth factor receptor 2 (HER2), BC is mainly divided into four different subtypes and treated accordingly [3, 4]. Luminal A (ER^+^/PR^+^/HER2^−^) and B (ER^+^/PR^−/low^HER2^−/+^) are the most frequent subtypes and represent around 60–70% of all BCs. These BC subtypes are sensitive to endocrine-based therapy through inhibiting estradiol (E2)/ER-mediated signaling, and clinical studies have shown that endocrine therapies can considerably reduce luminal BC recurrence and mortality [5]. However, up to 20% of the patients diagnosed with operable ER + tumors recur with metastatic disease, while endocrine resistance inevitably occurs in ER + metastatic or advanced BC [6, 7]. HER2^+^ BC is characterized with *HER2* gene amplification or protein overexpression, which accounts for around 20% of all BCs [4]. Humanized monoclonal antibodies and tyrosine kinase inhibitors, including trastuzumab, pertuzumab, pyrotinib and lapatinib, are clinically approved drugs to treat HER2^+^ BC [8, 9]. The introduction of these HER2-targeted drugs to the treatment of patients with HER2^+^ BC has led to dramatic improvements in survival in both early and advanced settings. However, nearly all patients with metastatic HER2^+^ BC eventually progress on anti-HER2 therapy due to de novo or acquired resistance [4]. Triple-negative breast cancer (TNBC: ER^−^/PR^−^/HER2^−^), a subgroup lacking the expression of hormone receptor (HR) and HER2, has no effective targeted therapy available like above-mentioned BC subtypes. Although TNBC patients are usually sensitive to chemotherapy, they are more prone to relapse and metastasize early and thus have a worse prognosis than other BC subtypes [3]. In addition, targeting immune checkpoints has shown therapeutic effect on improving the overall survival of TNBC patients with PD-L1 positive tumors [10]. However, these effects are only observed in patients with PD-L1 positive tumors. Therefore, identifying new therapeutic targets for treating TNBC is still an urgent demand.

nRTKs represent a large set of cytoplasmic tyrosine kinase family, which consist of ten members, including ABL, ACK, CSK, FAK, FES, FRK, JAK, SRC, SYK and TEC [11]. These kinases can be bound and activated by various RTKs, thereby regulating cancer development-related cellular events, such as cell polarity, proliferation, differentiation, migration, invasion and angiogenesis [11]. Among the nRTK family, SFKs are the most representative in mammals and are composed of eleven members, including BLK, BRK, FGR, FYN, FRK, HCK, LCK, LYN, SRC, SRM and YES [12]. Among these members, SRC is the first ever described tyrosine kinase proto-oncogene and also the most frequently implicated in tumorigenesis and metastasis of various cancer types, especially for BC [13]. Therefore, targeting SRC kinase represents an attractive strategy for BC therapy.

## SRC kinase-relevant functions in BC

**Fig. 1:**
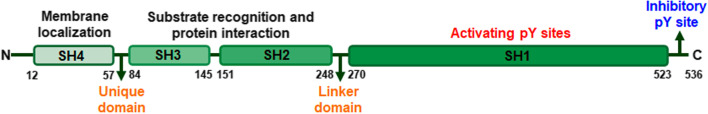
Schematic overview of the basic structure of SRC protein. SRC protein is mainly composed of seven parts (from N- to C-terminus): SH4 domain, unique domain, SH3 domain, SH2 domain, SH2-kinase linker domain, SH1 domain and the C-­terminal negative regulatory region

**Fig. 2:**
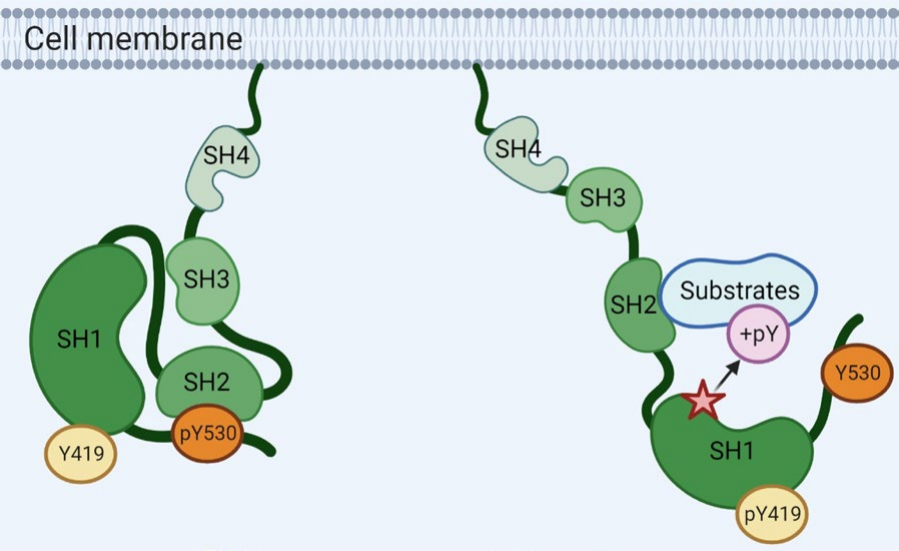
SRC kinase configuration with Tyr530 phosphorylation or Tyr419 phosphorylation, respectively. SRC activity is inhibited when the phosphorylated Y530 at the C­-terminal region binds to the SH2 domain, which will prevent the interaction of substrate proteins with the kinase domain (left panel). Dephosphorylated Y530 will induce the disassociation of the C-­terminal region from the SH2 domain, which allows substrate protein access to the catalytic kinase site in the SH1 domain, and be subsequently phosphorylated by SRC kinase (right panel)

The SRC protein is a 60-kDa protein tyrosine kinase [13]. Structurally, it mainly consists of seven parts from the N- to C-terminal: SRC homology 4 (SH4) domain, unique domain, SH3 domain, SH2 domain, SH2-kinase linker domain, SH1 domain and the C-­terminal negative regulatory region (Fig. 1). Among these domains, SH4 is responsible for SRC membrane localization; SH3 domain can bind to the proline-­rich peptides, thereby mediating the protein–protein interactions; the unique domain ling SH4 and SH3 domain usually varies among all SFK members; SH1 domain and the C-­terminal negative regulatory region contain tyrosine 419 and 530, respectively [14]. The phosphorylation of SRC Y530 residue by CSK or CHK will facilitate the interactions between SH2 domain and the C-­terminal regulatory region of SRC to form a pocket, thereby putting the kinase domain in this pocket and keeping SRC kinase in a closed configuration [15], while dephosphorylation of Y530 residue will display the substrate-binding pocket, allowing for the autophosphorylation at Y419 and the subsequent substrate accessing to the catalytic site (Fig. 2). SRC has been identified to be frequently overexpressed and/or aberrantly activated in various subtypes of BC, and high level of SRC activity is positively correlated with malignant potential and inversely correlated with the patient survival [13]. In this section, we will systematically summarize the functions of SRC that have been reported so far in different subtypes of BC.

### Luminal BC

The luminal subtype of BC (ER^+^/PR^±^HER2^−/+^) is characterized by the expressions of HR [7]. SRC has been shown to be essential for E2/ERα and progestin/PR-mediated signaling transductions, cell proliferation and cell cycle regulation [16]. Typically, in response to the stimulation of either E2 or progestin, ER or PR could directly interact with SRC kinase, leading to its relief from an intermolecular inhibitory conformation to an autoactivated form [17, 18]. Activated SRC could further initiate the Ras/Raf/MAPK cascade and induce BC cell proliferation [17, 18]. Reciprocally, the activated MAPKs could phosphorylate ER/PR and their binding factors, including STATs, to guide their downstream gene expression [19]. In addition, SRC-mediated ERα phosphorylation was revealed to be involved in the ERα interaction with its binding promoters, which was critical for ERα-dependent gene transcriptions and BC progression [16, 20].

Endocrine therapy is a crucial component of treatment for the luminal subtype of BC, which is usually performed either by inhibiting the production of estrogen or impeding the binding of estrogen to its receptors [21]. Some representative drugs, such as anastrozole and letrozole, are used to block the aromatase activity, thereby interfering with the androgen-converting into estrogen [7], while tamoxifen and raloxifene could compete with estrogen in binding ER to inhibit E2-mediated signaling transduction [7]. Although many patients have benefitted from endocrine therapy with a clear reduction in mortality and cancer recurrence, de novo and acquired resistance to this treatment remain a major challenge [7]. SRC activation has been considered as a survival signal for tamoxifen-resistant BC cells. Mechanistically, SRC-mediated MAPK signaling could induce ER phosphorylation and promote ER activation, as well as ER-regulated transcriptions in a ligand-independent manner [7, 22]. Meanwhile, multiple preclinical studies have reported that dual inhibition of SRC kinase and ER-mediated signaling can prevent acquired antihormone resistance in BC cells [23, 24]. In addition, ERα has been shown to directly interact with PI3K and SRC in a subset of invasive BC, and this complex thus represents a novel tumor biomarker to predict survival and/or response to targeted agents [25]. More interestingly, E2 can induce stress and apoptosis in long-term E2-deprived cells, while SRC activation has been revealed to play an essential role in mediating stress responses induced by E2 [26]. This study thus provided a mechanistic rationale for a new approach in the treatment of endocrine-resistant BC.

### ***HER2***^+^***BC***

Overexpression and/or amplification of *HER2/ERBB2/NEU* have been shown to be a causal factor for breast tumor malignancy and poor prognosis of patients [4]. Muller’s laboratory initially reported that elevated Src kinase activity was observed in *Neu*-induced mammary tumors [27]. Furthermore, disruption of Src kinase in this mammary tumor model could reduce the mammary tumor development [28]. These studies thus suggested that Src kinase was required for the induction of mammary tumors in transgenic mice. Subsequently, Tan et al. found that ErbB2-activated BC cells had higher metastatic potentials and increased Src activities compared with ErbB2 low-expressing cells [29]. Inhibition of Src activity significantly attenuated ErbB2-mediated cancer cell invasion in vitro and metastasis in an experimental animal model [29]. This study highlighted that increased SRC activities were required for ErbB2-mediated BC metastasis. A recent study further revealed that SRC activation could stimulate mitochondrial ATP production and suppress energy stress, which sustained the activation of mTORC1 and increased the translation of Ezh2 and Suz12, thereby driving ErbB2-related tumorigenesis and metastasis [30]. In addition, the clinical study also demonstrated that high levels of SRC activity in ductal carcinoma in situ were highly correlated with the clinicopathological factors, including HER2 status and the early recurrence [31]. Taken together, all these findings indicated the essential roles of SRC in the development, metastasis and prognosis of HER2^+^ BC.

Although trastuzumab has been demonstrated to effectively reduce the risk of recurrence and death in HER2^+^ BC patients, the majority of these patients possess de novo resistance or acquired resistance to trastuzumab during treatment [8, 9]. Zhang et al. have found that the BC patients with high SRC kinase activity are usually correlated with lower clinical response to trastuzumab-based therapy, higher progressive disease and shorter overall survival rates than patients having low SRC activity [32, 33]. Moreover, they also proved that SRC was a key modulator of trastuzumab response and a common node downstream of multiple trastuzumab resistance pathways, such as the activation of other RTKs and PTEN loss [33]. Combinational inhibition of SRC and HER2 activities reversed trastuzumab-resistant in vitro and eliminated tumors in vivo [34]. Therefore, inhibition of SRC-mediated signaling combining HER2-targeted therapy could be a very promising therapeutic strategy for patients with HER2^+^ BC.

### TNBC

Owing to lacking the expressions of ER, PR and HER2 in TNBC, targeted therapies are very limited for this subtype of BC. In addition, TNBC patients have a high incidence of early relapse and metastasis, with preferentially metastasizing to the bone, lung and brain [35]. Using a TNBC cell-based animal model, Myoui et al. have shown that Src kinase activity is positively correlated with the capacity of TNBC cells to develop bone and lung metastases [36]. Dasatinib is an orally active small molecule inhibitor targeting both SRC and other SFKs. Finn et al. have found that Dasatinib preferentially inhibits the growth of TNBC cell lines [37], and combining Dasatinib with several cytotoxic agents produces therapeutic synergy in preclinical TNBC models [38]. However, single-agent Dasatinib has very limited activity in unselected patients with TNBC [39].

Cancer stem-like cells (CSCs), a subpopulation of cancer cells that possess the ability to self-renewal and differentiation, have been proposed to contribute to the heterogeneity, relapse and therapy resistance of BC. BCSCs have been reported to be mainly enriched in TNBC cells, and targeting these cells thus becomes a priority for the development of novel therapy in TNBC patients [40]. Indeed, preclinical studies have demonstrated that a combination of Dasatinib and paclitaxel synergistically reduces TNBC cell viability in vitro and tumor growth in vivo [41, 42]. Utilizing chemotherapy-resistant TNBC patient-derived xenografts, Kohale et al. recently showed that treatment with Dasatinib led to the inhibition of tumor growth in vivo [43]. Therefore, these studies highlighted that targeting SRC-mediated BC stemness might represent an effective therapeutic regimen for TNBC.

## SRC kinase-mediated signaling transductions in BC

As a tyrosine kinase, SRC carries out its cancer-promoting functions mainly through catalyzing the tyrosine phosphorylation of various protein substrates. Therefore, identifying the key substrate of SRC in these processes will shed light on how these complexes contribute to the regulation of cellular events in BC. To this end, we aim to systematically summarize the SRC-mediated signaling transductions, with emphasis on its phosphorylation substrates in various contexts. We here have divided these substrates into three major groups according to their cellular localization to discuss their detailed biological functions in BC.

### Membrane targets in BC

**Fig. 3:**
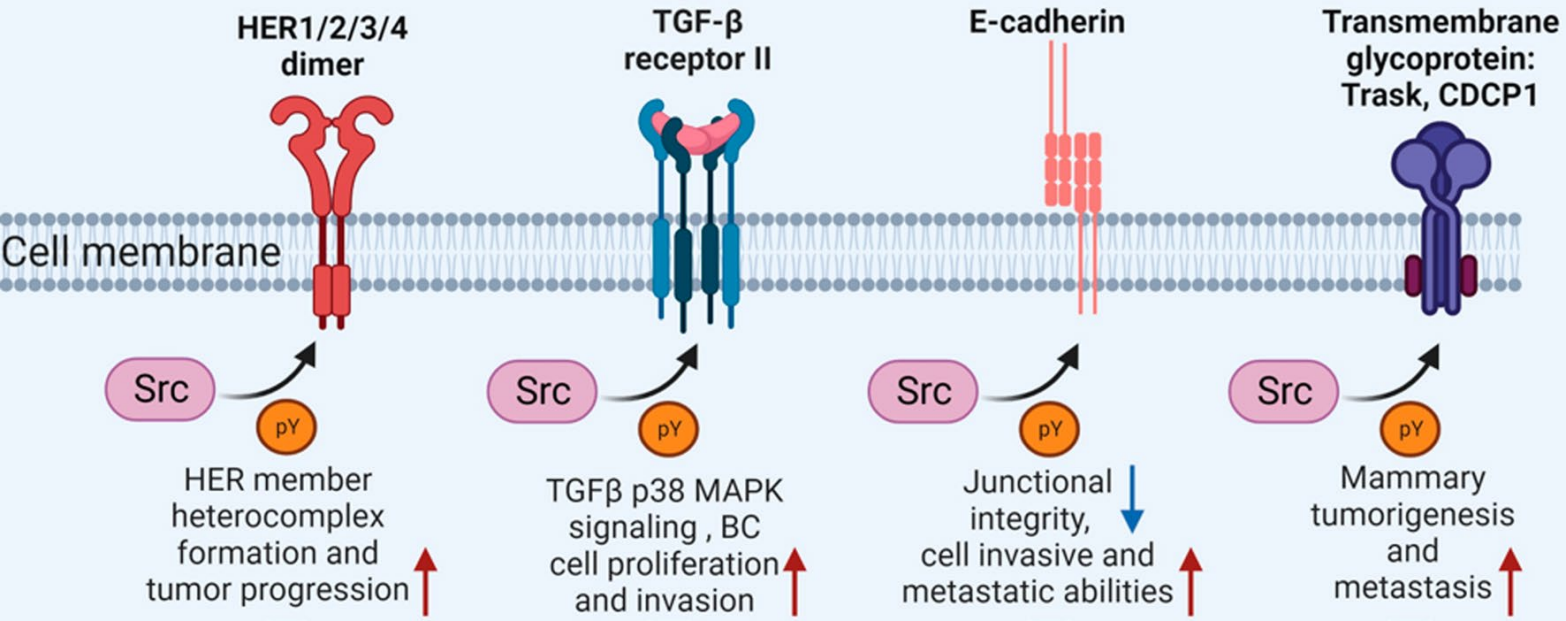
SRC kinase-mediated phosphorylation and function of membrane proteins in BC. SRC can interact with multiple membrane proteins through tyrosine phosphorylation, including HER family members, TGFβ receptor, AJ components and other transmembrane proteins, to coordinate the signaling transductions and cell proliferation, survival, migration, invasion and metastasis in BC

RTKs represent a large family of enzyme-linked receptors, which can be activated by ligand-mediated dimerization of kinases. The activated RTKs in turn phosphorylate specific tyrosine residues on the intracellular signaling proteins, to initiate a signal transduction cascade and gene expression. SRC is able to bidirectionally interact with multiple RTKs via its SH2 domain, thereby regulating cell proliferation and survival. Typically, these identified SRC-interacting RTKs include EGFR, vEGFR, PDGFR, FGFR and others [44] (Fig. 3 and Table 1). Among these SRC-associated RTKs, *EGFR* overexpression has been observed in a variety of cancer types, including BC. Specifically, SRC-mediated tyrosine phosphorylation of EGFR regulated its receptor function, as well as its oncogenic role in tumor progression [45]. Besides, in HER2^+^ BC, SRC-mediated ErbB2 phosphorylation could promote its oncogenic signaling by positively regulating ErbB2/ErbB3 heterocomplex formation [46]. In addition, TGFβRII is a serine/threonine kinase transmembrane receptor, its overexpression in mouse model enhances the mammary tumorigenesis [47]. Galliher et al. have shown that SRC-mediated tyrosine phosphorylation of TGFβRII facilitates the activation of TGFβ-p38 MAPK signaling, thereby promoting BC cell proliferation and invasion [48] (Fig. 3 and Table 1). Taken together, all these studies highlighted that targeting SRC kinase and these RTK activities might be efficient for treating SRC/RTKs-associated BC.

Adherens junctions (AJs) are described as the cell–cell connections between neighboring cells through direct interaction, which are mainly mediated by the cadherin–catenin protein complex. In mammals, AJs are essential for epithelial cell integrity, tissue formation and tumor suppression [49]. BC is mainly originated from the epithelial cells lining the ducts of the breast, and dysregulations of these AJs have been involved in the BC tumorigenesis and metastasis. SRC-mediated tyrosine phosphorylation of the E-cadherin/β-catenin complex in normal epithelial cells led to the loss of epithelial differentiation and induced the epithelial–mesenchymal transition (EMT) [50–52]. Besides, E-cadherin phosphorylation induced by SRC was also required for EGF-induced E-cadherin downregulation and AJ disassembly, as well as the acquisition of an invasive phenotype in breast tumors [53, 54] (Fig. 3 and Table 1). Trask is a 140-kDa type I transmembrane glycoprotein, which is also able to interact with Cadherin [55]. Trask is widely expressed in human normal epithelial tissues; however, its phosphorylation at tyrosine residues is observed in many epithelial tumors [56]. Further investigations revealed that SRC-mediated Trask phosphorylation was highly relevant to the mitotic regulation of cell adhesion and the epithelial tumorigenesis [56, 57]. CDCP1, another transmembrane glycoprotein overexpressed in BC, is a predictor of poor prognosis of patients [56]. Its phosphorylation by SRC was induced upon loss of cell adhesion and was thought to be linked to the metastatic potential of tumor cells [58, 59] (Fig. 3 and Table 1). These studies thus highlighted the central roles of SRC-dependent tyrosine phosphorylation in mediating AJ-associated tumor suppression, EMT and tumor metastasis in BC.

### Cytoplasmic targets in BC

**Fig. 4:**
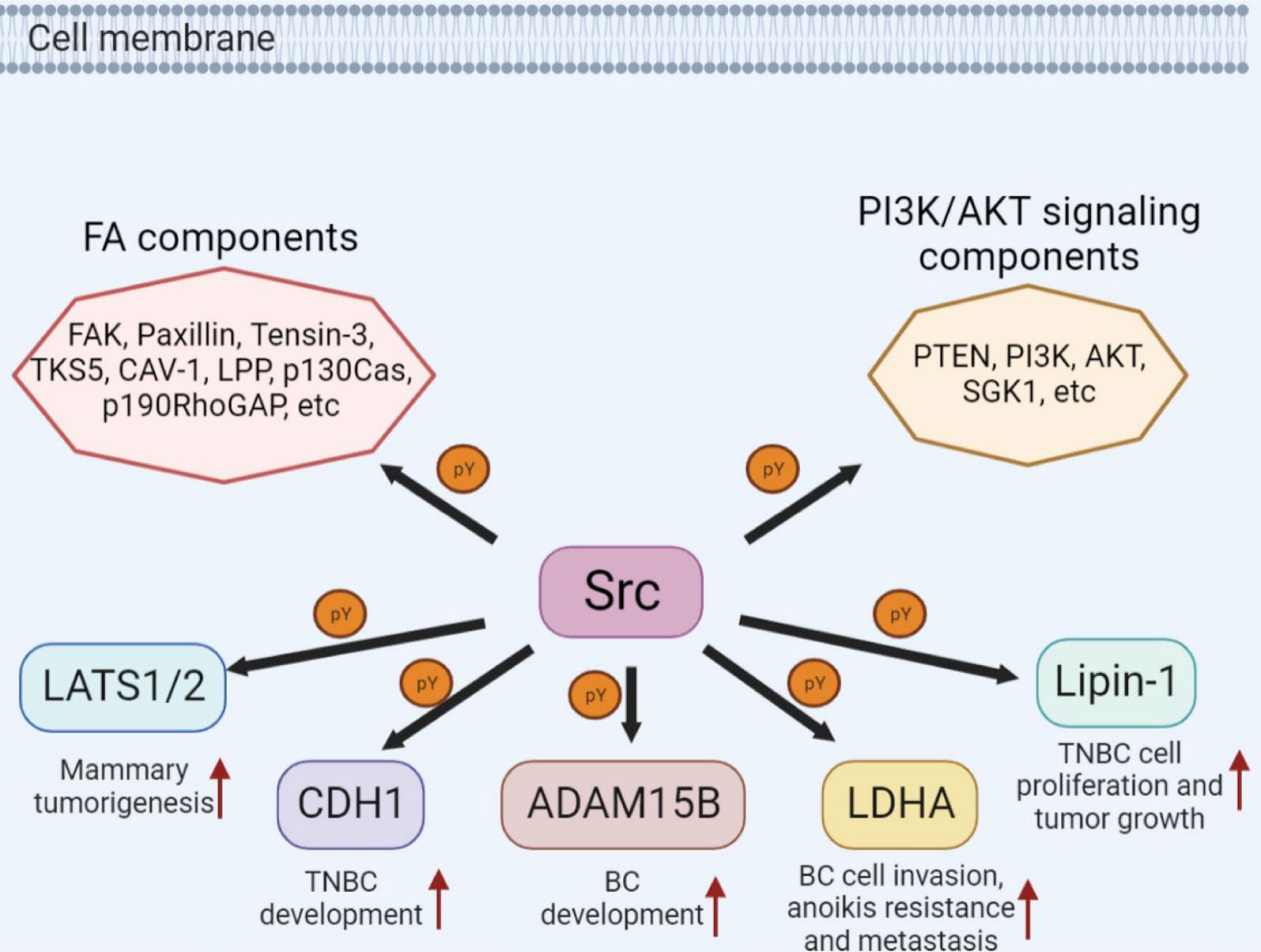
SRC kinase-mediated phosphorylation and function of cytoplasmic proteins in BC. SRC can phosphorylate multiple cytoplasmic proteins at tyrosine residues, including FA components, PI3K/AKT signaling components, as well as many protein kinases and proteases, thereby regulating mammary tumorigenesis and metastasis in various BC subtypes

Focal adhesion (FA) is defined as the cell attachment to the extracellular matrix (ECM) by integrins or intercellular transmembrane receptors, which connects the extracellular signals and the actin cytoskeleton. FA regulates a large number of integrin-mediated cell signaling events, including cell survival, proliferation, contraction, migration and differentiation. The composition of FA is quite dynamic and involved in various signaling, catalytic, cytoskeletal, adaptor and scaffold proteins. SRC-mediated tyrosine phosphorylation of integrin subunits can reduce the integrin binding strength to ECM, thereby promoting cell motility [60, 61]. For example, FAK is an nRTK, and it is also a major protein of the FA complex to mediate the integrin-mediated cell adhesion and migration [62, 63]. In BC, FAK activation has been shown to be required for ErbB2-mediated oncogenic transformation, invasion and tumor progression in vivo [64, 65], while SRC-mediated FAK tyrosine phosphorylation at multiple residues has been demonstrated to play an important role in full FAK activation [62, 63]. In addition, activated SRC/FAK module can further activate multiple other FA components to initiate a cascade of signal transduction events that regulate BC tumorigenesis and metastasis [66–68]. These FA components that have been reported in BC include Paxillin [69, 70], Tensin-3 [71], TKS5 [72], CAV-1 [73, 74], LPP [75], p130Cas [76] and p190RhoGAP [77–79] (Fig. 4 and Table 1). In addition, SRC kinase can also regulate the PI3K/AKT signaling through either inhibitory phosphorylation of PTEN [80] or activating phosphorylation of PI3K [81], AKT [33, 82, 83] or SGK1 [84], thereby regulating multiple events of BC development (Fig. 4 and Table 1). Altogether, these studies highlighted the essential roles of SRC-mediated tyrosine phosphorylation in FA regulation and PI3K-AKT signaling transductions, both of which were essential for SRC-induced mammary tumorigenesis and metastasis.

Besides FA and PI3K/AKT signaling components, many other cytoplasmic kinases or proteases were also identified to be directly phosphorylated by SRC kinase, thereby involving in the mammary tumorigenesis and progression (Fig. 4 and Table 1). For example, SRC-mediated LATS1 phosphorylation abolished the tumor suppressor activity of LATS1 and induced tumorigenesis in a YAP-dependent manner in BC cells [85, 86]. Similarly, SRC-mediated tyrosine phosphorylation of CDH1 could inhibit the ubiquitin E3 ligase activity of anaphase-promoting complex, thereby driving cell cycle progression and inducing mammary tumorigenesis [87, 88]. ADAM15B-mediated FGFR2 shedding has been implicated in the development of BC [89]. Maretzky et al. found that SRC-mediated ADAM15 phosphorylation was required for ADAM15 protease activity and the subsequent FGFR2 shedding [90]. In addition, SRC-related metabolic regulation was also found to be correlated with the invasive and metastatic potentials of BC cells. For example, LDHA is an enzyme that catalyzes the conversion of pyruvate and NADH to lactate and NAD^+^, and it is also a key step in glycolysis. Jin et al. have found that SRC kinase-mediated LDHA phosphorylation promotes BC cell invasion, anoikis resistance and tumor metastasis [91]. Phosphatidic acid phosphatase Lipin-1 is a lipid metabolism-related enzyme, which generates diglyceride precursors and is necessary for the synthesis of glycerolipids [92]. SRC-mediated Lipin-1 phosphorylation on multiple tyrosine residues could enhance its phosphatase activity, thereby promoting BC cell proliferation and malignancy [93]. These studies linked the SRC-mediated signaling with the metabolic alterations, which also represented an attractive point of therapeutic intervention for BC treatment.

### Nuclear targets in BC

**Fig. 5:**
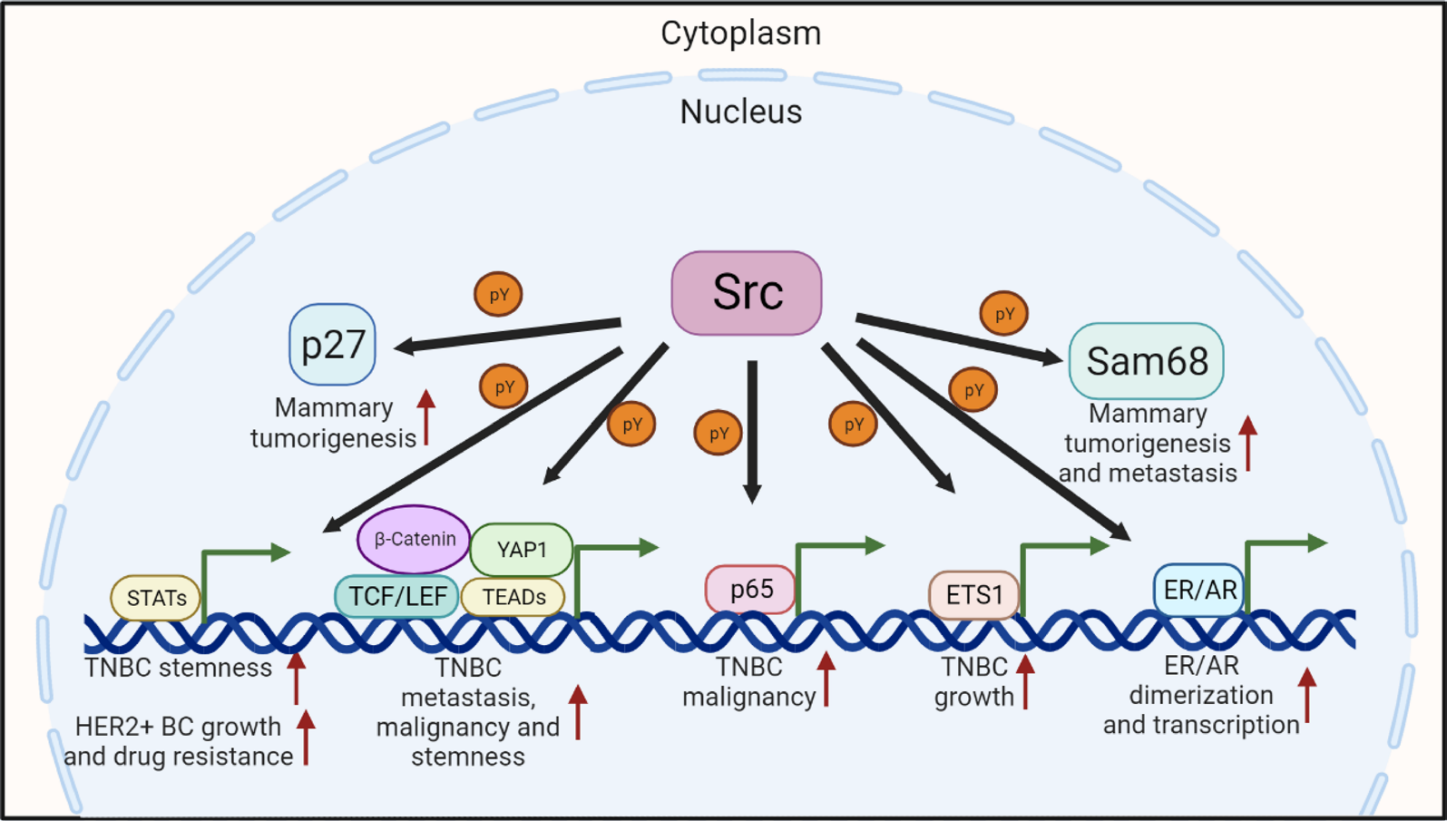
SRC kinase-mediated phosphorylation and function of nuclear proteins in BC. SRC can directly phosphorylate multiple nuclear proteins, including transcription factors, cell cycle regulators and RNA-binding proteins, thereby coordinating gene expression, cell cycle and BC-related cell behaviors

Multiple transcription factors or transcriptional regulatory proteins have been found to be directly activated by SRC in BC, including STATs, YAP1, NF-kB, etc. (Fig. 5 and Table 1). The STATs, such as 1, 3, 5a, 5b, were initially found to be activated by the intracellular JAK family kinases to mediate the cytokine-associated signaling transductions. Subsequent studies demonstrated that STATs could also be activated by a wide array of ligands and growth factors, including EGF, PDGF and some G-protein coupled receptor agonists [94]. Using both mouse fibroblast and human BC cell models, Garcia et al. and Kloth et al. have shown that the activation of STAT3/5 is responsible for BC tumorigenesis induced by the overexpression of SRC and EGFR. Specifically, SRC-mediated STAT3/5 phosphorylation enhanced their nuclear localization and binding to STAT-specific response elements, thereby inducing cell proliferation and survival [95, 96]. In addition, a recent study also revealed that SRC-mediated STAT3 signaling was required for the expression of pluripotency factors and BCSC enrichment in response to chemotherapy [97]. *NCAPG* expression is highly upregulated in trastuzumab-resistant HER2^+^ BC. SRC-mediated STAT3 nuclear localization and activation have been demonstrated to be responsible for NCAPG overexpression-induced trastuzumab resistance [98]. YAP1 and β-catenin are the downstream effectors of Hippo and Wnt signaling pathways, respectively, both of which participate in the occurrence and development of breast tumors. In RASSF1A-methylated BC tumors, Vlahov et al. have found that SRC-induced YAP1/β-catenin association through tyrosine phosphorylation is responsible for the *Myc* overexpression and invasive phenotypes of BC cells [52], while in BC-associated fibroblasts (CAFs), Calvo et al. have reported that the activation of YAP1 by SRC kinase is a signature feature of CAFs, which can further promote matrix stiffening, BC cell invasion and angiogenesis [99]. In addition, the activation of SRC by glucocorticoids-induced FA can increase YAP1 protein level, nuclear accumulation and transcriptional activity, thereby enhancing the CSC self-renewal and chemoresistance in basal-like BC subtype [100]. The latest study also showed that SRC-mediated β-catenin phosphorylation was responsible for EGF-induced aggressiveness and metastasis of TNBC cells [101]. Other transcription factors phosphorylated by SRC kinase, including NF-κB p65 and ETS1, are also essential for BC-associated phenotypes. Specifically, SRC kinase-mediated NF-κB activation is required for CTGF-induced *Glut3* expression and the aggressive phenotypes of TNBC [102], while SRC-mediated ETS1 phosphorylation can stabilize ETS1 and promote anchorage-independent growth in vitro and tumor growth in vivo [103]. Taken together, all these findings highlighted that SRC-mediated tyrosine phosphorylation on the transcription factors represented an important regulatory mechanism for BC tumorigenesis and development. Therefore, targeting the transcriptional outputs of these transcriptional factors in a specific context might be more straightforward for BC treatment.

Apart from the above-mentioned transcription factors, some nuclear receptors and nuclear-localized proteins, such as ER, AR, Sam68 and p27, were also found to be directly phosphorylated by SRC kinase (Fig. 5 and Table 1). Among these substrates, SRC-mediated ER phosphorylation is necessary for ER binding to the estrogen response element and its subsequent dimerization [104, 105]. While SRC-mediated AR phosphorylation is required for Kindlin-2-induced BC cell proliferation and migration in vitro and in vivo [106]. Based on these studies, the combination of endocrine therapy with SRC inhibitors may represent a treatment regimen in these subtypes of BC. In addition, the activities of cell cycle-associated protein and RNA-binding protein were also found to rely on SRC-mediated tyrosine phosphorylation. Typically, SRC-mediated p27 phosphorylation impairs the Cdk2 inhibitory action of p27 and thereby promotes the tamoxifen-resistance in BC cells, as well as the tumor progression in a mouse BC model [107, 108]. Sam68 is an RNA-binding protein, and SRC-mediated Sam68 phosphorylation has also proved to be necessary for mammary tumorigenesis and metastasis [109].

**Table 1:** SRC kinase-mediated signaling transductions in BC

Cellular localization	Substrates	Phosphorylated residues	BC subtypes and contexts	Functions	Main reference
Membrane	HER family proteins	Y845 and Y1101 of EGFR	BC cells	SRC-mediated phosphorylation of EGFR is required for its receptor function, as well as its oncogenic roles in tumor progression	[45]
	HER family proteins	Not reported	BC cells	SRC-mediated phosphorylation is required for ErbB2-mediated oncogenic signaling by positively regulating ErbB2/ErbB3 heterocomplex formation	[46]
	TGFβR II	Y284	BC cells	SRC-mediated tyrosine phosphorylation of TGFβRII facilitates the stimulation of TGFβ-p38 MAPK signaling, thereby promoting BC cell proliferation and invasion	[47, 48]
	E-cadherin	Y753, Y754 and Y755	Normal epithelial cells	SRC-mediated phosphorylation of E-cadherin disrupts the E-cadherin junctional integrity, and enhances the cell invasive and metastatic abilities	[50–52]
	E-cadherin	Y753, Y754 and Y755	TNBC	SRC-mediated phosphorylation of E-cadherin increases its internalization, subsequently destabilizing cell–cell junctions	[53, 54]
	Trask	Not reported	BC cells	SRC-mediated Trask phosphorylation is highly relevant to the mitotic regulation of cell adhesion and the epithelial tumorigenesis	[55–57]
	CDCP1	Y734, 743, 762, 707 and Y806	TNBC	SRC-mediated CDCP1 phosphorylation is linked to the metastatic potential of tumor cells	[56, 58, 59]
Cytoplasm	FAK	Y407, 576 577, 861 and Y925	BC cells and tumor models	SRC-mediated FAK phosphorylation is essential for full FAK activation, and FAK-mediated oncogenic transformation and invasion, and mammary tumor progression in vivo	[62–65]
	Paxillin	Y118 and Y31	BC cells and tumor models	SRC-mediated Paxillin phosphorylation regulates adhesion turnover, which is required for tumor cell invasion and metastasis	[69, 70]
	Tensin-3	Y1173, 1206 and Y1256	BC cells	SRC-mediated Tensin-3 phosphorylation contributes to the tumorigenesis and metastasis of BC cells and tumor	[71]
	TKS5	Y557 and Y619	BC cells	SRC-mediated TKS5 phosphorylation is required for the podosome formation and SRC-induced invasive phenotypes	[72]
	CAV-1	Y14	TNBC	SRC-mediated phosphorylation of CAV-1 is essential for its degradation and promoting BC cell stemness	[73, 74]
	LPP	Y245, 301 and Y302	HER2^+^	SRC-mediated LPP phosphorylation is critical for invadopodia formation, BC cell invasion and metastasis	[75]
	p190RhoGAP	Y1105	BC cells and tumor models	SRC-mediated p190RhoGAP phosphorylation regulates adhesion turnover, which is required for tumor cell invasion and metastasis	[77–79]
	PTEN	Y336	BC cells	SRC-mediated PTEN phosphorylation inhibits PTEN function and promotes the PI3K-AKT signaling cascade	[80]
	VPS34	Y231	BC cells	SRC-mediated VPS34 phosphorylation is required for the lipid kinase activity of VPS34 and SRC-induced cellular transformation	[81]
	AKT	Y315 and Y326	BC cells and tumor models	SRC-mediated AKT phosphorylation is essential for multiple evens of BC development and progressions	[33, 82, 83]
	SGK1	Not reported	TNBC	SRC-mediated SGK1 phosphorylation is required for SRC-mediated cell transformation in MCF10A cell	[84]
	LATS1	Y692 and Y916	BC cells	SRC-mediated LATS1 phosphorylation abolishes the tumor suppressor activity of LATS1 and induces tumorigenesis in a YAP-dependent manner in BC cells	[85, 86]
	CDH1	Y148	TNBC	SRC-mediated CDH1 phosphorylation could disrupt the interaction between Cdh1 and the APC core complex, and thus promote mammary tumorigenesis	[87, 88]
	ADAM15B	Y735	BC cells	SRC-mediated ADAM15 phosphorylation is required for ADAM15 protease activity and the subsequent FGFR2 shedding	[89, 90]
	LDHA	Y10	BC cells	SRC-mediated LDHA phosphorylation is required for cancer cell invasion, anoikis resistance and tumor metastasis	[91]
	Lipin-1	Y398, 413 and Y795	TNBC	Lipin-1 phosphorylation is required for SRC-enhanced glycerolipid synthesis, cell proliferation and xenograft growth in BC	[92, 93]
Nuclear	STATs	Y705 of STAT3 and Y699 of STAT5	EGFR/SRC overexpression	SRC kinase-mediated STAT3/5 activation is required for EGFR/SRC overexpression-induced BC tumorigenesis	[95, 96]
	STAT3	Y705 of STAT3	TNBC	SRC-mediated STAT3 signaling is required for the expression of pluripotency factors and BCSC enrichment in response to chemotherapy	[97]
	STAT3	Y705 of STAT3	NCAPG overexpression-associated HER2^+^	SRC kinase-mediated STAT3 activation is required for NCAPG-induced trastuzumab resistance in HER2^+^ BC	[98]
	YAP1 and β-catenin	Y357 of YAP1	RASSF1A-silenced BC	SRC kinase-mediated tyrosine phosphorylation of YAP1 and β-catenin is required for regulating the expression of β-catenin/TBX-YAP/TEAD target genes and the invasive phenotypes of TNBC	[52]
	YAP1	Y357	BC-associated fibroblasts	SRC kinase-mediated YAP function is required for BC-associated fibroblasts to promote matrix stiffening, cancer cell invasion and angiogenesis	[99]
	YAP1	Y357	TNBC	SRC kinase-mediated YAP tyrosine phosphorylation is required for glucocorticoids-induced stem cells traits in BC cells	[100]
	β-catenin	Y333	TNBC	SRC kinase-mediated β-catenin tyrosine phosphorylation is essential for EGF-induced aggressiveness and metastasis of TNBC cells	[101]
	NF-κB p65	Not reported	TNBC	SRC kinase-mediated NF-κB activation is required for CTGF-induced increase in Glut3 expression, glycolytic phenotype and aggressive phenotype of TNBC	[102]
	ETS1	Y283	TNBC	SRC-mediated ETS1 phosphorylation could stabilize ETS1 and promote anchorage-independent growth in vitro and tumor growth in vivo	[103]
	ER	Y537	Luminal	SRC-mediated ER phosphorylation is necessary for ER binding to the estrogen response element and the monomer to dimer transition	[104, 105]
	AR	Y534	TNBC	SRC-mediated AR phosphorylation is required for Kindlin-2-induced BC cell proliferation and migration in vitro and in vivo	[106]
	p27	Y74 and Y88	Tamoxifen-resistant BC	SRC kinase-mediated p27 phosphorylation impairs the Cdk2 inhibitory action of p27 and promotes the tamoxifen-resistance in BC	[107, 108]
	Sam68	Not reported	BC tumor model	SRC-mediated Sam68 phosphorylation is necessary for mammary tumorigenesis and metastasis	[109]

## SRC kinase-based target therapies in BC

Considering the dominant and broad roles of SRC kinase in mammary tumorigenesis and metastasis, SRC kinase inhibitors therefore hold great promise for the BC therapy. Multiple SRC kinase inhibitors have been previously developed by drug companies and approved by FDA for the treatment of hematologic tumors, including Bosutinib, Dasatinib and Saracatinib [12, 110]. Currently, these drugs are also widely used in the clinical trials for BC treatment. In this section, we mainly aim to summarize the clinical evidence and effects of SRC inhibitors as treatment in BC (Table 2).

Bosutinib is a multi-kinase inhibitor and has activity against all SFKs, as well as ABL. Multiple preclinical studies have demonstrated that Bosutinib can suppress BC cell growth, invasion and metastasis in vitro and in vivo [111, 112]. In addition, oral administration of Bosutinib in the MMTV-*PyVmT* transgenic mouse model could inhibit both the tumor initiation and tumor growth in older animals with preexisting tumors [113]. In a phase II clinical trial with metastatic BC patients, Bosutinib monotherapy showed a tolerable safety profile and moderated antitumor activity in a subset of patients with HR-positive BC [114]. However, the subsequent clinical trials combining Bosutinib with letrozole or exemestane in HR-positive BC patients did not receive a favorable risk–benefit profile with early termination of the studies [115, 116]. Besides, in a phase I study, Bosutinib combined with capecitabine demonstrated a safety profile; however, limited efficacy was observed in locally advanced/metastatic BC [117]. Therefore, further studies with Bosutinib in combination with other agents were warranted following the implementation of an appropriate method of patient selection. Beetham et al. recently revealed that loss of integrin-linked kinase activity can sensitize cells to Bosutinib treatment in a TNBC model [118], which may provide a new drug combination strategy for improving the clinical effectiveness of Bosutinib.

Dasatinib is an orally available small molecule targeting multiple SFKs, including SRC, LCK, FYN and YES [119]. Numerous in vitro and in vivo preclinical studies have demonstrated that Dasatinib has a high antitumor efficiency in various BC subtypes. However, clinical studies have confirmed that Dasatinib alone shows a very limited response when it is tested in TNBC and metastatic BC patients [39, 120–122]. To this end, a phase II study was designed to prospectively assess the utility of three previously published gene signatures to select patients with clinical benefits from Dasatinib [123]. Even so, none of these gene signatures could efficiently predict the clinical sensitivity to Dasatinib as a single agent. All these studies thus highlighted that Dasatinib has a very limited single-agent activity in unselected BC patients; further studies should consider Dasatinib combination with other agents in selected BC patients.

Multiple chemotherapeutic agents, such as paclitaxel and capecitabine, have shown great antitumor activity in both preclinical and clinical studies. Therefore, the combination of these agents and Dasatinib has been subsequently investigated in clinical trials to determine their synergistic antitumor activities. Typically, Fornier et al. showed that the combination of weekly paclitaxel and Dasatinib is feasible in phase I [124]; however, the phase II study of this combination is stopped early due to slow accrual [125]. Meanwhile, another phase II study with Dasatinib plus capecitabine shows a clinical benefit in 56% of response-evaluable patients with advanced BC, which supports further study with this combination in standard treatment [126]. Except for the combination of Dasatinib with chemotherapeutic agents, antihormone and HER2-targeted drugs are also widely used for evaluating efficacy and safety in combination with Dasatinib. Typically, in a non-comparative phase II trial, Dasatinib plus letrozole has shown efficiency in ER^+^/HER2^−^ metastatic BC, and this combination can delay the development of endocrine therapy resistance [127]. Additionally, SRC kinase is an essential factor for normal osteoclast function and for the development of bone metastases of BC [128]. A phase I/II study of Dasatinib in combination with zoledronic acid was designed to test their clinical efficacy in bone-predominant HER2-negative BC metastases. The result showed that this combination was well tolerated and potentially effective, owing to that a clinical benefit was observed for bone metastases in patients with HR-positive BC [129]. In HER2^+^ BC, phase I study has shown that the combination of Dasatinib with trastuzumab and paclitaxel is feasible, and shows a synergistic effect in patients with trastuzumab resistance [130]. Moreover, the phase II trial also showed this combination is active with an objective response rate of almost 80% in HER2^+^ metastatic BC patients [131]. Therefore, the combination of Dasatinib with trastuzumab and paclitaxel is highly recommended for the future clinical treatment of HER2^+^ metastatic BC patients. Taken together, all these studies indicated that combining Dasatinib with chemotherapy and other targeted drugs might be worth pursuing in molecularly determined patient subsets.

Saracatinib is an SRC-ABL kinase inhibitor. Compared to the Dasatinib, its adverse effects are moderate and easily managed. An early preclinical study has reported that Saracatinib and tamoxifen can cooperatively inhibit the growth of human ER^+^ BC cells [132]. In addition, combinational treatment of human BC cells using Saracatinib and tamoxifen can also effectively prevent antihormone resistance in vitro [23]. More importantly, Saracatinib markedly prevents the development of premalignant lesions and delays tumor onset in the MMTV-*Neu* mouse model [133]. Based on these studies, a phase II trial has been conducted to evaluate the efficacy and safety of Saracatinib monotherapy in unselected metastatic BC patients. However, the results are not sufficiently promising and Saracatinib does not show significant single-agent activity for the treatment of patients [134].

Compared to the above-mentioned SRC kinase inhibitors, the recently identified SRC inhibitor eCF506 has been proved to be more selective and specific for SFKs [110]. Moreover, eCF506 can reduce the TNBC cell growth in vitro and in vivo, as behaved like Bosutinib [118]. In addition, using a mouse TNBC metastasis model, eCF506 has mediated very potent in vivo antitumor activity against both primary tumors and bone metastases [135]. Based on these preclinical findings, eCF506 thus holds great promise as a first-in-class clinical candidate for the treatment of SRC-associated BC in the future.

## Conclusion and future perspectives

SRC is the first identified oncoprotein and also the first described protein tyrosine kinase. Over half a century of study has provided much information about its structure, function and SRC-mediated signaling transductions. Especially in human BC, SRC kinase is able to cooperate with multiple RTKs as well as a wide variety of downstream substrates, thereby regulating multiple events during tumor development. Despite that significant progress has been made in the elucidation of SRC-mediated signaling pathways, the translation from laboratory research to clinical application is not straightforward. The possible reasons may include: (1) *SRC* is rarely mutated or over-amplified like other oncogenes in BC, such as *EGFR* and *HER2*, which leads to the lack of a reliable predicative biomarker for response to SRC inhibitors. Therefore, a combination of the key downstream substrates in different contexts may be helpful for predicting the tumor development and utilizing the SRC inhibitors in clinic. For example, one recent study has revealed that Dasatinib radically reduced tumor growth in xenografts that have a signature of high pTyr characterization [43]. (2) Most of the SRC inhibitors in clinical testing are not selective and target other SFKs, which may have adverse events or side effects on both tumor cells and normal tissues. Therefore, elucidating the specific function of SRC in BC and developing more selective SRC inhibitors (like eCF506) may improve the clinical outcomes. (3) Due to the BC cell heterogeneity and its complex microenvironment, targeting SRC alone is very weak in clinic. Therefore, combinational treatment with SRC inhibitors and chemotherapeutics as well as other targeted drugs should continue to be explored in BC treatment clinical trials. Even more exciting is that the immunotherapy targeting immune checkpoint has been demonstrated to significantly improve the response to chemotherapy in PD-L1-positive metastatic TNBCs [10, 136, 137]. Therefore, finding more precise drug partners for SRC inhibitors is needed in the future.

**Table 2:** Clinical trials designed in breast tumors for the treatment with SRC kinase inhibitors

Drug name	Targets	Combination	Phase	Study purpose	Patient selection	Efficacy	Study outcome	Main reference
Bosutinib	All SFKs and ABL	/	II	To evaluate the toxicity and efficiency of Bosutinib	Locally advanced or metastatic BC pretreated with chemotherapy	PR = 5.5%, SD = 32.9%	Bosutinib showed promising efficacy and was generally well tolerated	[114]
		Exemestane	II	To evaluate the efficiency of Bosutinib plus exemestane as second-line therapy	Locally advanced or metastatic HR-positive/HER2-negative BC	PR = 2%, SD = 7%	An unfavorable risk–benefit profile was observed	115
		Letrozole	II	To evaluate the efficiency of Bosutinib plus letrozole as second-line therapy	Locally advanced or metastatic HR-positive/HER2-negative BC	PR = 6% SD = 6%	An unfavorable risk–benefit ratio was obtained	[116]
		Capecitabine	I	To evaluate the maximum tolerated dose, safety, and efficacy of Bosutinib plus capecitabine	Advanced/metastatic BC	/	Limited efficacy was observed	[117]
Dasatinib	SRC, LCK, FYN, and YES	/	II	To evaluate the efficacy and safety of Dasatinib monotherapy	Advanced HER2 + /ER + BC	PR = 4% SD = 13%	Limited single-agent activity was observed	[120]
		/	II	To assess the efficacy and safety of single-agent Dasatinib	Advanced TNBC	PR = 4.7% SD = 27.9%	Single-agent Dasatinib has limited activity in unselected patients with TNBC	[39]
		/	II	To evaluate efficiency and tolerability of Dasatinib combining with real-time pharmacodynamic tissue biomarkers	Metastatic BC	/	Single-agent Dasatinib did not exhibit significant antitumor activity in patients with metastatic BC	[121]
		/	II	To assess the efficacy of Dasatinib	Patients with bone-predominant BC metastasis	PR = 4%	Dasatinib was ineffective in controlling bone-predominant metastatic BC in a patient population unselected by molecular markers	[122]
		/	II	To assess the efficiency of Dasatinib combing with gene signature	Metastatic BC with predictive gene signatures	/	None of the predictive gene signatures could define tumor clinical sensitivity to Dasatinib as a single agent	[123]
		Paclitaxel	I	To determine the maximum tolerated dose of paclitaxel and Dasatinib	Metastatic BC	PR = 31% SD = 29%	120 mg daily (Dasatinib) and weekly paclitaxel were recommended	[124]
		Paclitaxel	II	To assess the efficiency of paclitaxel and Dasatinib	HER2-negative metastatic BC	PR = 20%	Study was stopped early due to slow accrual, and this combination showed some clinical activity	[125]
		Capecitabine	I	To assess the toxicity and maximum tolerated dose for Dasatinib plus capecitabine	Advanced BC	PR = 24% SD = 32%	The result supported further study with this combination in patients with advanced BC	[126]
		Letrozole	II	To assess the efficiency of aromatase inhibitor and Dasatinib	HR-positive metastatic BC	CBR = 71%	Letrozole plus Dasatinib was well tolerated	[127]
		Zolendronic acid	I/II	To determine the clinical efficacy of Dasatinib combined with zoledronic acid	Bone-predominant HER2-negative metastatic BC	CR + PR = 23% SD = 13%	Combination therapy was well tolerated and produced responses in bone in patients with HR-positive tumors	[129]
		Trastuzumab and paclitaxel	I	To assess the efficiency of trastuzumab plus paclitaxel in combination with Dasatinib	HER2-positive metastatic BC	/	This combination was feasible, and showed synergistic effect in patient with trastuzumab resistance	[130]
		Trastuzumab and paclitaxel	II	To assess the synergistic effect of Dasatinib and trastuzumab and paclitaxel	HER2-positive metastatic BC	PR = 69% SD = 10%	The combination was active with an objective response rate of almost 80%	[131]
Saracatinib	SRC and ABL	/	II	To evaluate the efficacy and safety of Saracatinib monotherapy	Unselected metastatic HR-negative BC	No response	Saracatinib did not show significant single-agent activity in HR-negative metastatic BC patients	[134]

*CR* complete response, *PR* partial response, *SD* stable disease, *CBR* CR + PR + SD

## Data Availability

The datasets used and analyzed in this study are available from the corresponding author upon reasonable request.

## Abbreviations

AJs: Adherens junctions
BC: Breast cancer
CAFs: Cancer-associated fibroblasts
CSC: Cancer stem-like cell
E2: Estradiol
ECM: Extracellular matrix
EMT: Epithelial–mesenchymal transition
ER: Estrogen receptor
FA: Focal adhesion
HER2: Human epidermal growth factor receptor 2
HR: Hormone receptor
MMTV: Mouse mammary tumor virus
nRTK: Non-receptor tyrosine kinases
PR: Progesterone
SFKs: SRC family kinases
SH: SRC homology
TNBC: Triple negative breast cancer
